# Effect of hyperthermic intrathoracic chemotherapy on the malignant pleural mesothelioma: a systematic review and meta-analysis

**DOI:** 10.18632/oncotarget.22062

**Published:** 2017-10-19

**Authors:** Zi-Yi Zhao, Sha-Sha Zhao, Meng Ren, Zi-Ling Liu, Zhi Li, Lei Yang

**Affiliations:** ^1^ Department of Orthopaedics, Dongzhimen Hospital, Beijing University of Chinese Medicine, Beijing 100700, China

**Keywords:** malignant pleural mesothelioma, hyperthermic intrathoracic chemotherapy, meta-analysis

## Abstract

Surgery-based multimodality therapies have been used to control the malignant effusion and its recurrence in malignant pleural mesothelioma (MPM). Hyperthermic intrathoracic chemotherapy (HITHOC) has been used in the treatment of malignant pleural mesothelioma, but the results were controversial. The aim of the current study was, therefore, to conduct a systematic review and meta-analysis on the effect of HITHOC on MPM therapy. After thorough searching of online databases, total 21 articles were included into qualitative systematic review and 5 of them were used to conduct qualitative meta-analysis. It was found that most of HITHOC was used in combination of surgical resection including extrapleural pneumonectomy or pleurectomy/decortication. Patients who received HITHOC had significantly longer median survival length compared to the patients without HITHOC (Hedges’s g = 0.384 ± 0.105, 95% CI: 0.178∼0.591, *P* < 0.001). In addition, HITHOC as palliative therapy was favored (Hedges’s g = 0.591 ± 0.201, 95% CI: 0.196∼0.967, *P* < 0.001) in terms of recurrence free interval. The findings of the current study suggested that HITHOC is one of the safe and effective therapies in prolonging patients’ median survival time and extending recurrence free interval.

## INTRODUCTION

Malignant pleural mesothelioma (MPM) is a fatal malignancy. Currently, median survival following diagnosis is often less than 12 months with limited options of therapies including surgery, radiotherapy and chemotherapy [1, 2]. Extrapleural pneumonectomy (EPP) has been widely used to treat early stage MPM and has been known to prolong survival time in patients with favorable prognostic factors [3, 4]. In addition, pleurectomy/decortication (P/D) has also been used in patients with MPM with or without radiotherapy and chemotherapy [5, 6]. However, significant proportion of patients have relapse of the disease following EPP or P/D and they usually die within a few months [7]. Thus, surgery-based multimodality therapies have been clinically explored in the past decades. In this regard, hyperthermic intrathoracic or intrapleural chemotherapy has been used as one of the multimodality therapies. Intrapleural injection of cytotoxic drugs with hyperthermic perfusion has been proved to enhance cytotoxic effect on tumor cells with limited systemic side effect. Potential mechanisms of hyperthermic intra-pleural or intraperitoneal chemotherapy are not only the tumor cells are directly exposed to higher concentration of chemotherapeutic agents, but also up to 44°C for 1 hr hyperthermic exposure render the cancer cells become more sensitive to the chemotherapeutic drugs while the normal tissues are unharmed [8, 9].

While cytoreductive surgery plus hyperthermic intraperitoneal chemotherapy (HIPEC) has become a standard therapy for intraperitoneal original carcinoma or carcinomatosis peritonei such as psudomyxoma and colorectal cancer induced ascites [10, 11], limited studies have been reported on the application of hyperthermic intrathoracic chemotherapy (HITHOC) in combination with surgery for the treatment of the malignant pleural effusion caused by variety kinds of tumors including mesothelioma, thyoma, breast cancer and lung cancer [12–14]. Especially, malignant pleural mesothelioma is highly aggressive and reports on the application of HITHOC in combination with EPP or P/D was controversial [15, 16]. The current review was, therefore, aimed to perform systematic review and meta-analysis on the publications of HITHOC application in the treatment of MPM. To accomplish this, online databases were searched and 21 articles were finally enrolled into the current study for systematic review and 5 articles were used for meta-analysis.

## MATERIALS AND METHODS

### Data sources

Relevant literature up to October 31st, 2016 was searched in the sites of PubMed, Embase and Web of Science with the following phrases: “hyperthermic intrapleural chemotherapy”, “intrapleural hyperthermic”, or “hyperthermic intrathoracic chemotherapy”, or “HITOC”, or “HITHOC” and “mesothelioma”. The search was limited to English and Chinese, and relevant studies were also identified by hand-searching the references.

### Inclusion criteria

Studies were included into the current systematic review and meta-analysis if: 1). Clinical studies on the treatment of malignant pleural mesothelioma (MPM) with hyperthermic intrapleural or intrathoracic lavage and/or chemotherapy; 2). Studies with full text articles.

### Data extraction

Data extraction was conducted by Sha-Sha Zhao and Fu-Jun Han. Data extraction included study name (the first author’ last name), year of publication, treatment regimen, total number of cases for each treatment group, median survival months, one-year survival rate, 2-year survival rate, 5-year survival rate, recurrence free interval, and morbidity of adverse effect of HITHOC therapy.

### Statistical analysis

The following format of data entry was used: 1). Median survival month of overall survival or disease free survival, number of cases, and *P* value; 2). Median survival month of recurrence free interval, number of cases, and *P* value. The strength of HITHOC therapeutic effect on MPM was measured by Hedges’s g. A fixed effect model was applied when no heterogeneity was observed among the studies. Alternatively, a random effect model was applied if the heterogeneity between studies was *P* < 0.10 and I^2^ > 50%, which was considered as heterogeneous between the studies [17]. All meta-analysis was performed using the Comprehensive Meta-analysis software (Version 3, NJ, USA).

## RESULTS

### General information of the enrolled studies

**Figure 1:**
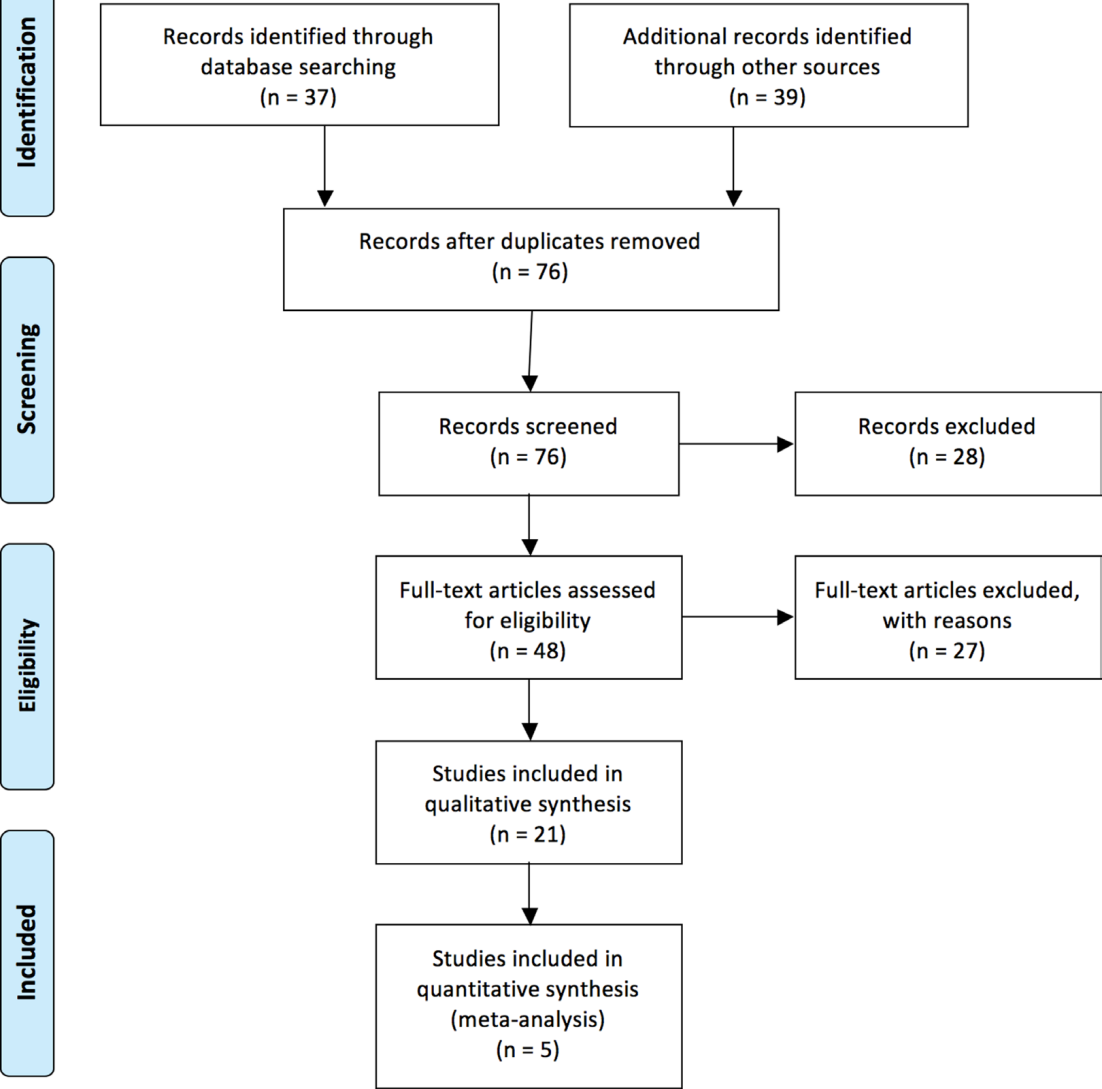
Flow chart of database search and literature selection

As shown in Figure 1, after careful reading of the abstracts, total 48 full-text articles were retrieved. The articles were then independently assessed and data was extracted by two investigators (Meng Ren and Zi-Ling Liu). After excluding reviews and case report articles, total 21 articles were included in the systematic review [6, 14, 18–31] and 5 articles were included in the meta-analysis [15, 16, 32–34]. Of the 21 articles for systematic review and meta-analysis, 5 articles were from USA [6, 16, 20, 21, 32]; 4 articles were from Netherlands [14, 15, 18]; 3 articles were from United Kingdom [22, 23, 33]; 2 from Japan [24, 25], 2 from Italy [30, 31], 2 from Germany [26, 27], one from Israel [28], Turkey [34], and France [29], respectively.

Most commonly used agent for hyperthermic intrapleural chemotherapy was cisplatin, and the temperature applied for hyperthermic chemotherapy in most of the mesothelioma was between 38–43°C (Table 1).

**Table 1:** Summary of the data extraction for systematic review

Author’s Name	Year	Country	Treatment	Drugs	Case of MPM	MST (m)	RFI (m)	OSR	Morbity	Reference
Rusch	1994	USA	P/D + HITHOC	CDDP	28	17		1 yr: 68%; 2 yr: 40%		6
Yellin	2001	Israel	EPP + HITHOC	CDDP	7			1 yr: 57%		28
de Bree	2002	Nethelands	CRS + HITHOC		11			7 m: 82%	47%	14
Monneuse	2003	France	Surgery + HITHOC	CDDP	17	T1 & T2: 41.3		1 yr: 74%		29
				Mitomycin C		T3&T4: 4.5		5 yr: 27%		
van Ruth	2003	Nethelands		Doxorubicin	24	Pharmakinetics				19
				CDDP						
van Ruth	2003	Nethelands	CRS + HITHOC	CDDP	22	11		1 yr: 42%		18
				Doxorubicin						
Richards	2006	USA		Low dose: CDDP	9	6	4		9∼32%	20
				High dose: CDDP	35	18	9			
Xia	2006	Japan	RT + HITHOC	CDDP/CBDCA	11	27.1				24
Lang-Lazdunski	2011	UK	P/D + HITHOC	CDDP	36	24		1 yr: 91.7%	3.9∼13.9%	22
				Pemetrexed				2 yr: 61%		
Sugarbaker	2012	USA	P/D+HITHOC	Mitomycin C	25	Pharmakinetics				21
				Doxorubicin	4					
Ried	2013	Germany	P/D + HITHOC	CDDP	8	18		22 m: 50%		27
Ried	2013	Germany	P/D + HITHOC	CDDP	10	Pharmakinetics				26
Ishibashi	2015	Japan	P/D + HITHOC	CDDP	4		23∼41	2 yr DFS: 75%		25
			EPP + HITHOC	CDDP	10	12.1		2 yr DFS: 27%		
Lang-Lazdunski	2015	UK	P/D + RT + HITHOC	CDDP	102	32		5 yr: 23.1%	29.40%	23
Migliore	2015	Italy	Debulking + HITHOC	CDDP	6	13.6				30
Migliore	2015	Italy	P/D + HITHOC	CDDP	6	21.5			16.60%	31

CDDP:cisplatin; CBDCA: carboplatinum;DFS: disease free survival; EPP: extrapleural pneumonectomy; HITHOC: hyperthermic intrathoracic chemotherapy; MPM: malignant pleural mesothelioma; MST: median survival time; OSR: overall survival rate; P/D: pleurectomy/decortication; RFI: recurrence free interval; RT: radiotherapy

### Effect of hyperthermic intrapleural chemotherapy on survival

Of the 21 articles selected for systematic review, 5 studies were compared the therapeutic results of the patients received hyperthermic intrapleural chemotherapy during cytoreductive surgery or extrapleural pneumonectomy to that of the patients without hyperthermic intrapleural chemotherapy [15, 16, 32–34], 14 studies were retrospective studies on the effect of HITHOC on mesothelioma without comparison to non-HITHOC treatment [6, 14, 18, 20, 22–26, 28–31], 3 studies reported pharmacokinetics of chemotherapeutic drugs in the pleural cavity [18, 21, 26].

**Figure 2:**
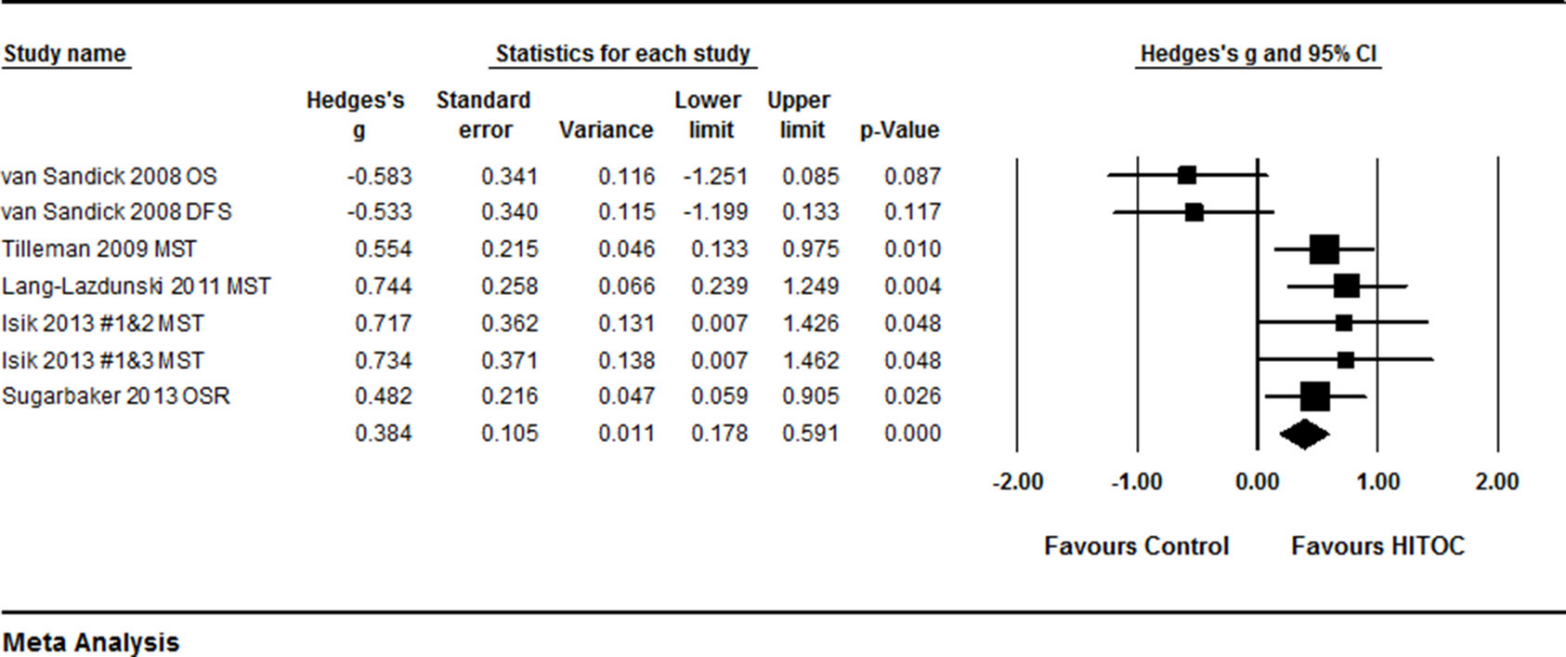
Forest plot for median survival time of MPM patients with or without HITHOC A random effect model was used due to non-significant heterogeneity of publications (I^2^ = 70.76, *P* = 0.002). Effect size was assessed by Hedges’s g and 95% CI, and the median overall survival (OS) or disease free survival (DFS) time was in favors HITHOC (Hedges’s g = 0.384 ± 0.105; 95% CI: 0.178∼0.591, *P* < 0.001). Isilk #1: patients treated with HITHOC following surgical intervention; Isilk #2: patients treated with talc pleurodesis followed by systemic treatment; Isilk #3: patients treated with pleurectomy/decortication followed by systemic treatment.

**Figure 3:**
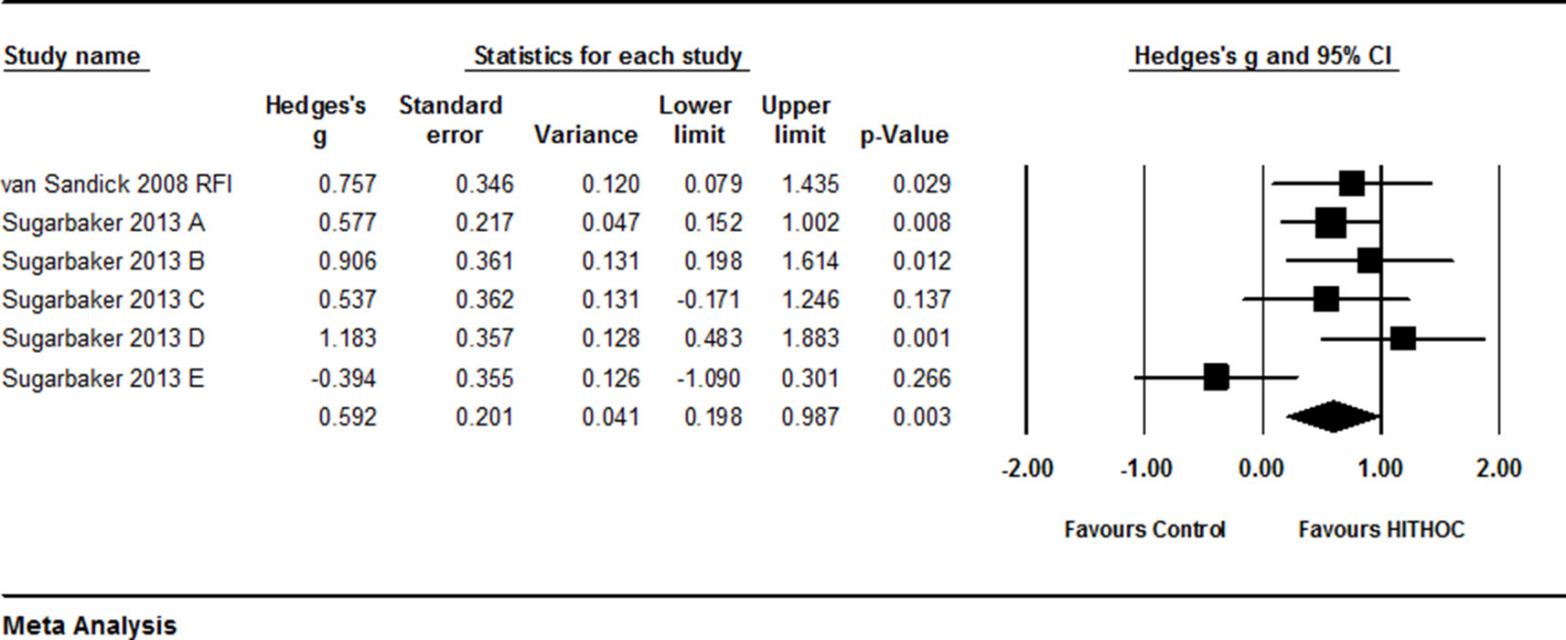
Forest plot for recurrence free interval (RFI) for patients with or without HITHOC A random effect model was used due to significant heterogeneity of publications (I^2^ = 56.4, *P* = 0.043). Effect size was assessed by Hedges’s g and 95% CI, and the RFI was in favor of HITHOC therapy (Hedges’s g = 0.591 ± 0.201, 95% CI: 0.196∼0.967, *P* < 0.001). Sugarbaker (A) RFI in all patients; Sugarbaker (B) RFI in the patients without radiotherapy; Sugarbaker (C) RFI in the patients with radiotherapy; Sugarbaker (D) RFI in the patients with N1 or N2 lymph node metastasis; Sugarbaker (E) RFI in the patients in N0 status.

By quantitative meta-analysis of the 5 studies [15, 16, 32–34], it was found that average of the median survival time was significantly longer in the patients treated with HITHOC compared to the patients without HITHOC (Hedges’s g = 0.384 ± 0.105, 95% CI: 0.178∼0.591, *P* < 0.001, Figure 2, Table 2) although the study of van Sandick et al from Netherlands reported that median survival time of overall survival and disease free survival was longer in the patients treated with extrapleural pneumonectomy (EPP) and postoperative hemithoracic radiation (RT) compared to the patients treated with EPP and intraoperative HITHOC [15]. In addition, HITHOC therapy was favored in terms of mesothelioma recurrence free interval time (Hedges’s g = 0.591 ± 0.201, 95% CI: 0.196∼0.967, *P* < 0.001, Figure 3). Furthermore, Tilleman et al reported a phase II prospective study [32]. They reported that total 96 of 121 (79%) enrolled patients underwent EPP, of whom 92 (76%) received hyperthermic intraoperative intrapleural cisplatin perfusion after EPP. The median overall survival of the 121 enrolled patients was 12.8 months, median survival of the 92 patients treated with HITHOC was 13.1 months, which was significantly longer than that of the 29 patients without hyperthermic intrapleural cisplatin perfusion (11.0 months, *P* = 0.01).

**Table 2:** Summary of the data extraction for meta-analysis

Author’s Name	Year	Country	HITHOC				Non-HITHOC		Reference
			Total #	MST (m)	RFI (m)	Total #	MST (m)	RFI (m)	
van Sandick	2008	Netherlands	20	OS: 11	9	15	OS: 29	19	15
				DFS: 8			DFS: 21		
Tilleman	2009	USA	92	13.1		29	11		32
Lang-Lazdunski	2012	UK	54	23		22	12.8		33
Sugarbaker	2013	USA	72	35.3	27.1	31	228	12.8	16
Isik	2013	Turkey	19	15.4		13	6		34

DFS: disease free survival; HITHOC: hyperthermic intrathoracic chemotherapy; MST: median survival time; OSR: overall survival rate; RFI: recurrence free interval

All of the 16 clinical studies without proper controls demonstrated that hyperthermic intrapleural chemotherapy could significantly prolong patients’ life and improve quality of life. In this regard, recently, Lang-Lazdunski et al [23] reported 102 patients (73 of them, 71.5%, were mesothelioma) with malignant pleural effusion were treated with pleurectomy/decortication plus hyperthermic pleural lavage with povidone-iodine. They found that overall median survival and 5-year survival rate was 35 months and 30.7% for epithelioid mesothelioma, and 15 months and 7% for non-epithelioid mesothelioma. Ried et al. [27] reported that four patients with malignant pleural mesothelioma were treated with cytoreductive surgery and hyperthermic intrathoracic chemotherapy perfusion. They found median survival was 18 months and two of them were with no evidence of mesothelioma till the analysis. Monneuse et al. reported that the 1-, 2-, 3-, and 5-year actuarial survival rates were 69, 50, 42 and 5%, respectively, in the patients with mesothelioma who received surgery plus intrathoracic chemohyperthermia [29].

### Side effects of hyperthermic intrapleural chemotherapy

Most of the 21 articles enrolled into the current systematic review and meta-analysis reported no perioperative or HITHOC-associated (30-day or 90-day) mortality. However, various morbidity of adverse effect following the surgery plus hyperthermic intrapleural chemotherapy had been reported. The rate of morbidity was between 5.3∼65%. Specifically, Tilleman et al. reported that, out of the 92 patients, 22 (23.9%) patients had atrial fibrillation, 12 (13.0%) had thrombosis/thrombus/embolism, 10 (10.9%) had laryngeal nerve dysfunction, 6 (6.5%) had acute respiratory distress syndrome/respiratory failure [32]; Yellin et al. reported that 4 out of 26 patients (15.4%) had empyema after HITHOC with cisplatin [28]; De Bree et al. reported that 2 out of 14 patients (14.3%) had diaphragm rupture [14]; Richards et al. reported that, out of 44 patients, 14 (32%) had atrial fibrillation, 4 (9%) had deep venous thrombosis, 5 (11%) had respiratory failure or adult respiratory distress syndrome, 25 (57%) had renal toxicity [20]. Most recently, Liu et al. from China has reported safety issue of intrapleural hyperthermic chemotherapy [35]. They reported that overall intrapleural hyperthermic chemotherapy-associated mortality in 1,510 times of hyperthermic intrathoracic chemotherapy for 315 cases of malignant pleural effusion was zero. Nevertheless, overall morbidity of adverse effect was 2.0%, specifically, 0.6% pneumothorax, 0.3% cytotoxic agent-induced pleural inflammation, and 0.5% pain at puncture location [35].

### Pharmacokinetics of locally administrated cytotoxic agents

Only limited number of published data is available on the pharmacokinetic features of intrapleural administered cytotoxic drugs and its systemic impact in malignant pleural mesothelioma. In this regard, Ried et al. performed pharmacokinetic analysis of intrapleural cisplatin with a two different dosages (100 mg/m^2^ and 150 mg/m^2^) at 42°C perfusate temperature [26]. They found: 1). Area under the curve (AUC) ratios of perfusate versus serum were nearly similar in the two different dosages and lasted up to 24 hrs after perfusion; 2). The mean AUCs of cisplatin in the perfusate were approximately 58 and 55 times greater than detected in the serum; 3). The mean peak of cisplatin in the serum was reached after 1 hr of hyperthermic intrapleural chemotherapy [26].

Sugarbaker et al. compared pharmacokinetics of intrathoracic hyperthermic chemotherapy (HITHOC) versus that of intraperitoneal hyperthermic chemotherapy(HIPEC) [21]. They found: 1). Approximately 41 ± 3 percent of the total mitomycin C was absorbed from the thoracic space into the body compartment during the 90 minutes HITHOC; 2). The amount of mitomycin C absorbed from the pleural space was approximately half of the amount absorbed from peritoneal space. Moreover, there was also a considerably more rapid clearance from the abdomen as compared to that from the thorax [21].

## DISCUSSION

The optimal treatment of malignant pleural mesothelioma (MPM) relies on not only surgical resection, but also other multimodality therapies including radiotherapy and hyperthermic intrapleural chemotherapy. MPM is an aggressive malignancy and has high recurrence rate. One of the main objectives for pulmonary oncologists is to prolong recurrence free interval as well as overall and disease free survival time. In this regard, hyperthermic intrathoracic chemotherapy (HITHOC) has recently been applied as a palliative treatment for MPM during or after surgery. Reports on the therapeutic effect of HITHOC on MPM, however, were controversial. Therefore, in the current study, we systematically reviewed and performed meta-analysis on 21 articles to determine the effect of HITHOC on controlling progress and recurrence of MPM. Through the systematic review and meta-analysis of the selected 21 papers, we found that HITHOC significantly prolonged median survival time of the MPM patients who received HITHOC in addition to surgical resection including extrapleural pneumonectomy (EPP) and pleurectomy/decortication (P/D).

Currently, EPP or P/D surgery is the major therapy for MPM. Other therapies including radiotherapy or intrapleural chemotherapy with or without hyperthermic perfusion were also used intra-operatively or post-operatively in order to enhance therapeutic effect as well as to prevent recurrence of the disease. Most of the 21 articles included in the current study reported that HITHOC as one of the multimodality therapies could prolong patients’ median survival time although one study indicated that HITHOC had no advantage in the treatment of malignant mesothelioma [15]. Through the meta-analysis of the five studies enrolled into this study, we further demonstrated that MPM patients benefited from the HITHOC treatment when they were given HITHOC either intra-operatively or post-operatively. Meta-analysis results of the five studies also indicated that combination of surgical resection and HITHOC, which allowed direct delivery of the cytotoxic agent to the tumor cells, significantly extended the recurrence free interval of MPM.

Most popular cytotoxic drugs used for HITHOC were cisplatin followed by doxorubicin and mitomycin C, and 41–43°C was most commonly used in HITHOC. In theory, hyperthermia can improve the efficacy of chemotherapy by increasing local drug absorption and enhancing chemotherapeutic drug action [36]. The mechanism of hyperthermia is thought to be protein denaturation of the cancer cells at temperature up to 44°C for 1 hour while the normal tissues are unharmed at this temperature [37]. Protein denaturation of cancer cell leads to an increase in the rate of tumor cell apoptosis through affecting cell membrane cytoskeleton, DNA synthesis and membrane permeability [8]. In addition, intrapleural chemotherapy allows for a much higher concentration of the drugs in the thoracic cavity compared to systemic chemotherapy, thereby improving cytotoxicity to the tumor cells and minimizing systemic adverse effect. Pharmacokinetic studies of the cytotoxic drugs used for HITHOC indicated that a persistently high concentration of intrapleural drug was achieved when the patients were given HITHOC although the absorption efficiency of intrapleural delivery was approximately half of that of intraperitoneal delivery [21].

While surgical procedure of EPP or P/D on MPM may cause morbidities of adverse effects such as bronchopleural fistula, diaphragm rupture or laryngeal nerve dysfunction, HITHOC *per se* did not cause such adverse effects. In this regard, most recently, Liu et al. from China has reported that overall HITHOC-associated morbidity of side effects in 1,510 times of hyperthermic intrapleural chemotherapy for 315 cases of malignant pleural effusion was 2.0% in overall, that was, 0.6% pneumothorax, 0.3% cytotoxic agent-induced pleural inflammation, and 0.5% pain at puncture location [35]. In addition, perioperative or 30-day post-operative mortality following HITHOC was zero [23, 35]. These findings suggested that HITHOC is relatively safe and tolerable to the patients. Furthermore, Liu et al. reported a procedure of bedside HITHOC [35], and they used local anesthesia and puncture technology to establish a sealed hyperthermic perfusion circulation. By using this technique and device, the patients remained conscious during the whole HITHOC procedure. This was superior to conventional HITHOC performed during surgery or post-operation with systemic anesthesia in that the latter may cause problems such as increased intrathoracic and central venous pressure, hemodynamic alterations and the risk of systemic hyperthermia [38].

There are several limitations in the current systematic review and meta-analysis. The major limitation is that only 5 studies are eligible for the meta-analysis and the number of cases included in each study is small. Second, HITHOC was applied in combination with other multimodality therapies in all of the 5 studies enrolled into this study. Third, techniques of HITHOC used in the 21 articles were heterogeneous including difference of cytotoxic drug and their concentration, equipment used for HITHOC, volume and temperature of the perfusion solution, and perfusion time etc. Fourth, the “control” group in the 5 selected studies for meta-analysis was not properly controlled and randomized. Fifth, effect of radiotherapy or systemic chemotherapy subsequent to surgical resection of MPM was not compared with that of HITHOC due to the limited number of studies. Thus, it is in urgent situation to standardize the method of HITHOC in the treatment of MPM. Nevertheless, findings of the current systematic review and meta-analysis indicate that HITHOC is an effective and safe therapeutic modality for prolonging patient’s life and extending recurrence free interval of the disease.

Taken together, EPP or P/D plus hyperthermic intrathoracic chemotherapy significantly prolong patients overall survival time and extend recurrence free interval of the patients MPM. HITHOC as one of the surgery-based multimodality therapies for MPM is effective and safe.
